# Commentary: Education for automation

**DOI:** 10.1155/S1463924679000353

**Published:** 1979


### Commentary

### Education for automation

Many superlatives can be used to describe the new microcomputer-controlled analytical/clinical instrumentation. It is certainly apparent that the new technology has opened the door for powerful automated systems that can expedite research, greatly improve routine analytical determinations, and revolutionize methods for operating a laboratory. Thus, both the retraining of personnel and new courses for science students become extremely important.

Courses and conferences on microprocessors,microcomputer applications, programming, interfacing, and digital/analog electronics are available, and these are providing an important background for a small percentage of chemists. What seems to be missing is an integrated course – a course that provides both an overall "working ability" with today's devices and automated techniques and an awareness of the automated systems that could be introduced tomorrow. If the stage is truly set for automated laboratories and automated research, and if sophisticated laboratory robots are getting ready in the wings, then our training programmes should be geared for these dramatic changes.

Two decades ago we introduced a new type of integrated course to teach electronics and new measurement concepts to already-trained scientists and advanced-level science students. New types of laboratory training devices were devised, experiments tested, and a text written so as to provide an overall experience that would solve some of the problems of that period. Problems of how to put a new instrument into operation, how to keep it going, how to modify it for a new research problem, how to increase sensitivity or eliminate noise, how to prevent inaccuracies because of interaction between the measurement device and the system under test, and how to mechanize or automate a process or analytical method. In short, a specific course of study and experimentation was devised that enabled the typical scientist to experience a breakthrough of the "electronics barrier" in a minimum amount of time. These materials were updated regularly as the new digital and microelectronic devices appeared on the scene. Both a very intensive 3-week course for already-trained scientists and a one-semester course for advanced-level college students proved successful, and were used for training purposes in many industries and colleges throughout the world. No similar course of study and experimentation exists so that a typical scientist can experience a breakthrough of the "automation barrier" in a minimum amount of time.

It is true that in recent years many "patchboard" experimental systems and texts have been developed for teaching certain aspects of digital and analog electronics and microcomputers. However, no elegant integrated package of text, experiments, and experimental devices has yet become available to the scientific community. A powerful blend of digital and analog electronics (including modern servo systems), computerization, interfacing, measurement principles, and vision is needed. It will again be necessary to develop laboratory training systems and state-of-the-art experiments so that chemists and other scientists will readily gain exciting first-hand experience with today's automated systems, as well as a "feel" for what could be done–a future awareness.

It is hoped that at least one or more of the trial programmes now underway at various institutions will soon be distilled into a first-rate programme that will bear much fruit. It must be remembered that concentrating so much on a single integrated course does not mean that one is "getting something for nothing". Rather, an all-out effort is required. The important point is that with a carefully designed course the material can be absorbed, and absorbed effectively, in a relatively short period. The principles and concepts of automation, the computer-managed laboratory, laboratory robots, remote labs, and automated research centres can and should soon be integrated into a course that will become readily accessible to scientists and students.

*Howard V. Malmstadt*

### From the Editor's desk

Professor Malmstadt's commentary focuses attention on one of the most vital issues relating to automation at the present time, that is education. In such a rapidly expanding and progressing arena it is vitally important for us to quickly communicate the important advantages and philosophies inherent in their introduction. It becomes increasingly necessary to understand new disciplines and to talk across interdisciplinary boundaries.

In this issue Professor Browner, of Georgia Technical Institute, gives a brief overview of the approach he has taken to pass on the philosophy of automation to graduate students working towards a PhD degree. This goes some way to meeting Professor Malmstadt's points, but perhaps it is wrong to expect any one school to be able to cover all such parameters. What is needed is a concerted effort on behalf of a group of people covering many interests to co-ordinate views and design a suitable course structure.

The Summer School on automation organised by the Chemical Society at Swansea, UK, 8 - 13th July 1979, is an experiment conducted to meet some of the needs in education. Details of this course are described in this issue (page 160). However, a course of this nature, of a single week's duration, will only provide an overview of the problem and will offer little "hands-on" experience so necessary to provide a feel for the subject. At least it will open the attendee's minds to new technology and persuade them to rethink their approaches to the analytical methods they use with automation in mind.

As mentioned earlier, there is a real need to co-ordinate views and philosophies of automation; it was to this end that the *Journal of Automatic Chemistry* was conceived. It is difficult for various groups of workers with interests in automation to meet and exchange views. With the exception of the Technicon Symposia, which are necessarily restricted in their coverage, there is no international meeting of note which focusses its attention on this problem. Attempts have been made to co-ordinate a European interest in automation but despite efforts to spark off a steering committee to meet and discuss possible co-operative meetings no progress has been made.

In the UK the Automatic Methods Group of the Chemical Society Analytical Division acts as a co-ordinating body for industrial automation applications but discovering who if anyone carries out a similar function elsewhere is difficult to discern. Clinical chemists are infinitely more organised in this respect so it is possible, and vital,for interests among practising automation exponents to be organised.

Analysis 1979 is devoted to Automation in Industrial and Clinical Chemistry (see page 108 of the January issue) and offers an ideal catalyst for such discussion on a European scale. I am sure that time can be found within this meeting for interested people to meet and exchange views on the problem and also to discuss the needs with clinical chemists also attending the meeting. I personally would welcome any interest in this and will attempt to co-ordinate a meeting if

